# CTHRC1 overexpression predicts poor survival and enhances epithelial‐mesenchymal transition in colorectal cancer

**DOI:** 10.1002/cam4.1807

**Published:** 2018-10-09

**Authors:** Shujuan Ni, Fei Ren, Midie Xu, Cong Tan, Weiwei Weng, Zhaohui Huang, Weiqi Sheng, Dan Huang

**Affiliations:** ^1^ Department of Pathology Fudan University Shanghai Cancer Center Shanghai China; ^2^ Department of Oncology Shanghai Medical College, Fudan University Shanghai China; ^3^ Institute of Pathology Fudan University Shanghai China; ^4^ Wuxi Cancer Institute Affiliated Hospital of Jiangnan University Wuxi China

**Keywords:** colorectal cancer, CTHRC1, epithelial‐mesenchymal transition, prognosis, TGF‐β

## Abstract

Collagen triple helix repeat containing (CTHRC1), which was identified as a cancer‐related factor, is a promigratory protein involved in multiple processes, including vascular remodeling, antifibrosis, metabolism, bone formation, and cancer. In this study, we aimed to investigate the clinical significance and possible role of CTHRC1 in the process of epithelial‐mesenchymal transition (EMT) in colorectal cancer (CRC). Here, we revealed that CTHRC1 mRNA and protein levels are both upregulated in CRC tissues compared with those of paired noncancerous tissues. Moreover, the overexpression of CTHRC1 correlated with poor prognosis in patients with CRC (especially colon cancer). Furthermore, we showed that CTHRC1 induced EMT and promoted cell motility in CRC cells. Importantly, we demonstrated that CTHRC1 promoted EMT by activating transforming growth factor‐β (TGF‐β) signaling, revealing a possible effective therapeutic treatment for patients with CRC.

## 1 INTRODUCTION

Colorectal cancer (CRC) is the third most common cause of cancer deaths in China^1^ and the second most common cause of cancer deaths worldwide.^2^ Although a multidisciplinary approach to treating CRC is evolving, the prognosis of patients with late‐stage CRC remains very poor.^3^ Clinically, distant metastasis and recurrence are responsible for most cancer‐related deaths. However, precise prediction and targeted therapy for metastatic tumors are unavailable in the clinic because the molecular mechanisms that underlie metastatic spread remain largely unclear.

Several processes are involved in the occurrence and metastasis of CRC, including epithelial‐mesenchymal transition (EMT).^4^, ^5^, ^6^ EMT is defined by the loss of an epithelial phenotype and the acquisition of a motile, invasive, and migratory mesenchymal phenotype. EMT is a complex multistep process that requires initiating signals to drive transition. Increasing evidence suggests that signal transduction pathways, such as the transforming growth factor (TGF‐β) pathway, are key modulators of the EMT process.^7^, ^8^

Collagen triple helix repeat containing 1 (CTHRC1) was first discovered in balloon‐injured rat arteries, where it is involved in vascular remodeling and promoting cell migration.^9^, ^10^ In cancerous tissue, CTHRC1 was overexpressed in most solid tumors,^11^, ^12^, ^13^, ^14^ including CRC.^15^, ^16^ However, the underlying mechanism of CTHRC1 activation is largely unknown. In vascular cells, CTHRC1 regulated TGF‐β responsiveness^9^ and reversed TGF‐β‐stimulated collagen expression.^17^ Due to the modulation of TGF‐β in the EMT process during cancer cell migration, we proposed that CTHRC1 might be involved in TGF‐β ‐related EMT and CRC metastasis.^8^

To identify the potential role of CTHRC1 in CRC progression, we analyzed the correlations between CTHRC1 and both clinicopathologic variables and outcomes. Then, we investigated whether CTHRC1 could promote cancer cell migration and invasion through the EMT process in CRC cells. To explore the precise molecular pathways that are driven by CTHRC1, we examined CTHCR1 function in CRC cell lines and its potential involvement in TGF‐β‐signaling‐induced EMT progression.

## 2 MATERIALS AND METHODS

### 2.1 Patients and follow‐up

This study was performed in accordance with local ethical and legal requirements after approval by the Ethics Committee of Fudan University Shanghai Cancer Center. A total of 216 patients were diagnosed and underwent surgical resection for primary CRC between April 2012 and December 2016. Medical records were reviewed for clinical information, and histologic parameters were evaluated from hematoxylin & eosin (H&E) stained slides. Tumor budding was measured in accordance with the International Tumor Budding Consensus Conference (ITBCC).^18^ The count of 0‐4 buds was classified as Bd1, 5‐9 buds as Bd2 and 10 or more buds as Bd3. Between January and April 2017, all patients were followed up by telephone or mail to determine patient survival. A data set from *The Cancer Genome Atlas* (TCGA) and *The Human Protein Atlas* (THPA) were utilized for the evaluation of CTHRC1 mRNA and protein expression in CRC (Supporting Information).

### 2.2 Real‐time quantitative reverse transcription polymerase chain reaction

RNA extractions were performed using an RNeasy Mini kit (Qiagen, CA, USA). Real‐time quantitative reverse transcription polymerase chain reaction (RT‐qPCR) experiments were conducted using a Premix Ex Taq Real‐Time PCR kit (Toyobo, Osaka, Japan) on an ABI 7500 cycler, with β‐actin as an internal control. Relative mRNA expression was calculated using the delta‐delta Ct method, and control cells were used as calibrators. Detailed primer information is listed in Table S1.

### 2.3 Tissue microarray

The tumor microarrays were constructed using paraffin‐embedded, formalin‐fixed tissues from 216 CRC specimens and 33 adjacent normal colorectal tissues (mucosa from the resection margin) with a tissue arrayer (Beecher Instruments, Silver Spring, MD) as previously described. For each case, three core samples were acquired from normal and tumor blocks, and tissue cores were 1.0 mm in diameter.

### 2.4 Immunohistochemistry and scoring

Standard immunohistochemistry (IHC) staining was performed using the avidin‐biotin immunoperoxidase technique with the following primary antibodies: an anti‐CTHRC1 antibody (rabbit polyclonal antibody against CTHRC1, 1:100 dilution; from Abcam, Cambridge, MA, USA) and an anti‐pSmad2 antibody (rabbit polyclonal antibody against CTHRC1, 1:100 dilution; from Abcam).

The annotation process included an estimation of the intensity of immunoreactivity (negative: 0; weak: 1; moderate: 2; and strong: 3) and the fraction (%) of positive cells (0, <5%; 1, 5%‐25%; 2, 25%‐50%; 3, 50%‐75%; and 4, >75%). The H‐score, which was defined as the sum of the product of the staining intensity (0‐3) multiplied by the percentage of positive cells (0‐4), was calculated. All tumors were categorized as low CTHRC1 protein expression (score ≤6) or high CTHRC1 protein expression (score >6). All staining slides were reviewed by two experienced and independent pathologists.

### 2.5 Cell culture and treatment

Colo‐205, DLD‐1, HCT‐8, HCT116, Lovo, and SW480 colon carcinoma cells were acquired from the Pathology Lab of the Fudan University Shanghai Cancer Center and validated by short tandem repeat (STR) profiling.^19^ Cells were grown in culture according to standard procedures as described in the Supplementary Materials and Methods.

Recombinant TGF‐β (PeproTech, Rocky Hill, NJ, USA) was added to the culture medium at a concentration of 50 ng/mL. TGF‐β signaling was inhibited using SB‐431542 (a transforming growth factor receptor type I (TGFRI) kinase inhibitor) diluted in DMSO (Sigma‐Aldrich, Louis, MO, USA). DMSO served as control medium in all experiments.

### 2.6 Plasmid construction and transfection

CTHRC1 cDNA was amplified by PCR using the following primer pair: 5′‐GCTAGCATGCGACCCCAGGGCCCCG‐3′ (F) and 5′‐ CTCGAGTTATTTTGGTAGTTCTTCAATAAT‐3′ (R). The PCR product was cloned into the pIRES2‐EGFP vector (BD Biosciences, San Jose, CA, USA). DLD‐1 cells were transfected with the pIRES2‐EGFP‐CTHRC1 or pIRES2‐EGFP vector using the Lipofectamine 2000 transfection reagent (Invitrogen, Carisbad, CA, USA) according to the manufacturer's instructions.

### 2.7 RNA interference

A small interfering RNA (siRNA) assay was used to knock down CTHRC1 in CRC cells using the Lipofectamine 2000 transfection reagent (Invitrogen). A CTHRC1‐specific siRNA (5′‐GACCUGUAUAAUGGAAUGUTT‐3′) was synthesized by Invitrogen.

### 2.8 Migration and invasion assays

Cell migration and invasion assays were performed as described in Supplementary Materials and Methods.

### 2.9 Western blot analysis

Western blot was performed using standard procedures as described in Supplementary Materials and Methods. The primary antibodies included anti‐CTHRC1 (Abcam, ab85739, 1:100), anti‐α‐catenin (Sigma‐Aldrich, C2081, 1:1000), anti‐E‐cadherin (Dako, M3612, 1:2000), anti‐fibronectin (Abcam, ab2413, 1:2000), anti‐vimentin (Dako, M0725, 1:5000), and anti‐β‐actin (Sigma‐Aldrich, A2066, 1:4000).

### 2.10 Immunofluorescence staining

Cells cultured on coverslips were washed with phosphate‐buffered saline (PBS), fixed with 4% paraformaldehyde, permeabilized with 0.1% Triton X‐100, and blocked with 1% bovine serum albumin (BSA). The cells were then stained with an anti‐E‐cadherin antibody (Dako, 1:200), followed by the appropriate anti‐mouse Alexa Fluor 488‐conjugated secondary antibodies (BD Biosciences). The samples were mounted using ProLong Gold Antifade Mountant with DAPI (Invitrogen), and fluorescence was visualized with a confocal microscope (Leica Microsystems, Bensheim, Germany).

### 2.11 Luciferase reporter gene assays

Luciferase reporter gene assays were conducted using the SBE‐LUC Reporter Kit (BPS Bioscience). Quantification of firefly luciferase was performed using a Dual‐Luciferase Reporter Assay System (Promega Co, Madison, WI, USA). All plasmids (50 ng) were transfected into cells in triplicate using Lipofectamine 2000 (Invitrogen). Thirty‐six hours after transfection, the cells were treated with 50 ng/mL TGF‐β for an additional 12 hours.

### 2.12 Statistical analysis

All data analyses were conducted using SPSS 22.0 statistical software. The Wilcoxon test was performed to compare the levels of gene expression in CRC and paired adjacent normal tissues. Relationship analysis between categorical values was performed using the chi‐square test. Disease‐free survival (DFS) and overall survival (OS) curves were calculated using the Kaplan‐Meier method and analyzed with the log‐rank test. DFS was calculated from the date of surgery to the date of progression (local and/or distal tumor recurrence) or the date of death. OS was defined as the length of time between diagnosis and death or last follow‐up. Univariate and multivariate survival analyses were performed using a Cox proportional hazards regression model. The significance tests were two‐sided, and a *P* value <0.05 was considered statistically significant.

## 3 RESULTS

### 3.1 CTHRC1 is related to the clinical aggressiveness of CRC

**Figure 1:**
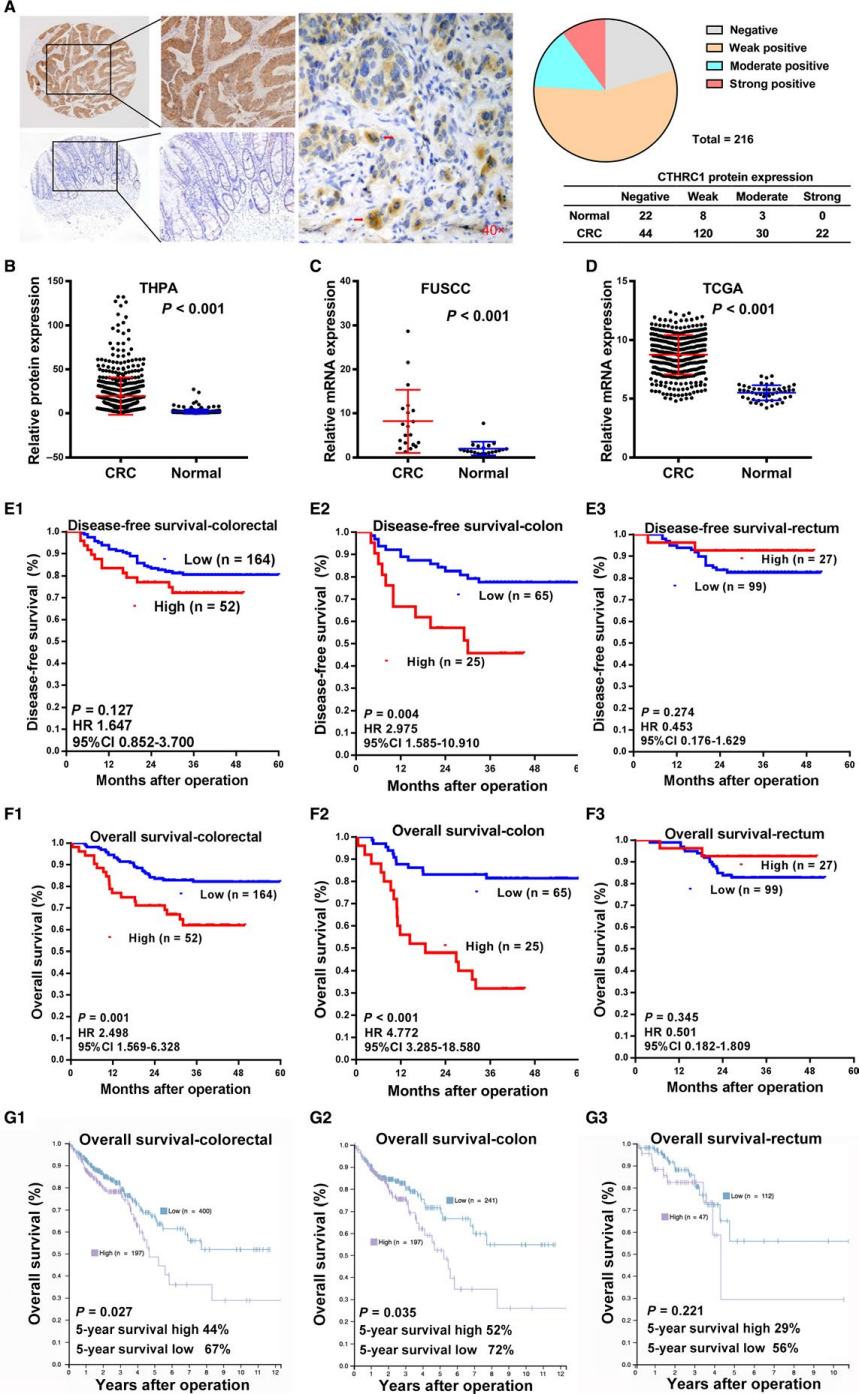
CTHRC1 expression in human CRC. A, Representative immunohistochemical analysis of CTHRC1 in CRC (upper left) and normal colorectal mucosa (lower left) samples. CTHRC1 staining was increased in tumor buds of cancerous tissues (middle, red arrows). Pie chart presenting the CTHRC1 staining groups in CRC (n = 216) and normal mucosa samples (n = 33). B, Analysis of THPA data indicating elevated CTHRC1 protein expression in 597 CRC samples compared to that of adjacent normal tissue samples (196 transverse mucosa and 149 normal sigmoid mucosa samples) (mean ± SD, *P* < 0.001 with one‐way ANOVA). C, Relative mRNA expression of CTHRC1 was detected by RT‐qPCR in 20 pairs of primary CRC samples and adjacent colorectal mucosa samples. β‐actin was used as an internal control. The data are presented as the mean ± SD and compared by the Wilcoxon test. D, Analysis of TCGA data indicating that CTHRC1 expression is elevated in colorectal cancers (n* = *224) compared with that of normal colorectal tissues (n* = *22) (mean ± SD, with an unpaired *t* test). E, Kaplan‐Meier survival curve with log‐rank analysis of DFS according to the CTHRC1 expression in total CRC (E_1_), colon cancer (E_2_), and rectal cancer (E_3_). CTHRC1 expression was associated with DFS in colon cancer (E_2_), but not in total CRC (E_1_) or rectal cancer (E_3_). F, Kaplan‐Meier survival curve with log‐rank analysis of OS according to the CTHRC1 expression in total CRC (F_1_), colon cancer (F_2_), and rectal cancer (F_3_). CTHRC1 expression was associated with OS in total CRC (F_1_) and colon cancer (F_2_), but not in rectal cancer (F_3_). G, Kaplan‐Meier survival curves with log‐rank analysis of the colorectal cancer data in the THPA data set. Increased CTHRC1 mRNA expression was associated with poor OS in patients with CRC (G_1_) and colon carcinoma (G_2_), but not rectum carcinoma (G_3_)

To evaluate the clinical significance of CTHRC1 expression, tissue microarrays with 216 CRCs and 33 adjacent normal mucosa were subjected to CTHRC1 IHC staining (Table 1). Strong or moderate staining was seen in 52 CRC samples, which showed cell membrane and cytoplasmic immunopositive for CTHRC1 antibody (Figure 1A). With respect to the H‐score calculations, the rate of high CTHRC1 expression (score >6) in CRC samples was 24.1% (52/216) and that rate was significantly lower in normal tissues (9.1%, 3/33). The data for CTHRC1 protein expression from the THPA database also indicated that CTHRC1 was highly expressed in CRCs compared with the level in normal colon samples (*P* < 0.001, Figure 1B). Similarly, PCR was used to evaluate CTHRC1 mRNA levels in cancer samples in 20 pairs of primary CRC tissues and adjacent colorectal mucosa samples (*P* < 0.001, Figure 1C). Consistent with these data, the CTHRC1 mRNA expression data from the TCGA colorectal database showed that the mRNA levels of CTHRC1 were significantly higher in CRC samples than those in normal tissues (*P* < 0.001, Figure 1D). In addition, high expression of CTHRC1 was associated with advanced stage (*P* = 0.016) and tumor budding (*P* = 0.022, Table 1). Especially, the increased CTHRC1 staining was observed in the advancing front within tumor buds (Figure 1A, arrows).

**Table 1:** Relationship between CTHRC1 expression and clinicopathologic parameters of colorectal cancer patients

Characteristics	Number of case	CTHRC1 expression				*P* value
		High (n = 52)	%	Low (n = 164)	%	
Age (y)	216					
<50	46	19	36.5	27	16.5	0.187
≥50	170	33	63.5	137	83.5	
Gender						
Male	130	30	57.7	100	61.0	0.746
Female	86	32	42.3	64	39.0	
Tumor size						
<5 cm	122	34	65.4	88	53.7	0.151
≥5 cm	94	18	34.6	76	46.3	
Location						
Colon	90	25	48.1	65	39.6	0.333
Rectum	126	27	51.9	99	60.4	
Histologic grade						
Well	10	2	3.8	8	4.9	0.101
Moderate	182	40	76.9	142	86.6	
Poor	24	10	19.2	14	8.5	
Depth of invasion						
T1	2	1	1.9	1	0.6	0.463
T2	29	5	9.6	24	14.6	
T3	185	46	88.5	139	84.8	
Lymphatic metastasis						
Absent	106	20	38.5	86	52.4	0.083
Present	110	32	61.5	78	47.6	
Venous invasion						
Absent	196	48	92.3	148	90.2	0.788
Present	20	4	7.7	16	9.8	
Nervous invasion						
Absent	210	50	96.2	160	97.6	0.632
Present	6	2	3.8	4	2.4	
Tumor budding[Link]						
Bd1&Bd2	114	63	54.8	51	45.2	**0.022**
Bd3	102	31	29.6	71	70.4	
Distant metastasis						
Absent	177	40	76.9	137	83.5	0.303
Present	39	12	23.1	27	16.5	
Duke's stage						
I and II	94	15	28.8	79	48.2	**0.016**
III and IV	122	37	71.2	85	51.8	
Recurrence[Link]						
Absent	164	36	69.2	128	79.0	0.062
Present	50	16	30.8	34	21.0	

According to Ref. 18

Analyzed with Kaplan‐Meier method and the log‐rank test.

Statistical significances (*P* value < 0.05) marked in bold font.

### 3.2 Association of CTHRC1 expression with the prognosis of CRC

**Figure 2:**
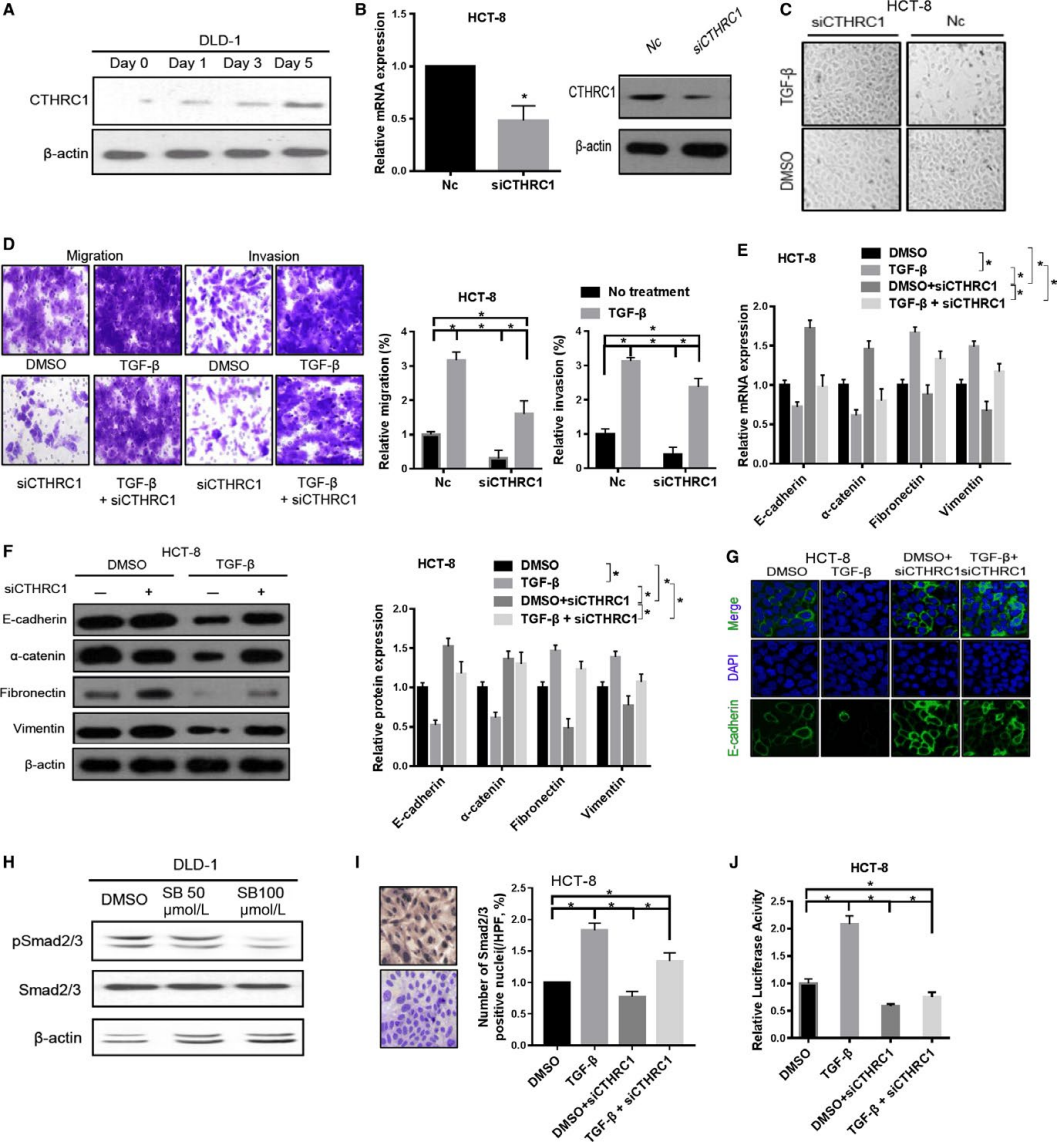
CTHRC1 is involved in TGF‐β signaling. A, TGF‐β induces CTHRC1 protein expression in DLD‐1 cells. The loading amount was normalized to the level of β‐actin. B, Knockdown of CTHRC1 in HCT‐8 cells with siRNA was confirmed by RT‐qPCR and Western blotting. β‐actin was used as an internal control. The data were compared by a paired *t* test (*P < 0.05). C, EMT‐like morphological changes induced by TGF‐β treatment were markedly blocked by CTHRC1 knockdown in HCT‐8 cells. D, CTHRC1 knockdown hampered the TGF‐β‐induced migration and invasion of CRC cells. The data were compared by an unpaired *t* test (*P < 0.05). E, Effects of CTHRC1 knockdown on the mRNA expression of EMT markers in HCT‐8 cells treated with DMSO or TGF‐β. The data were compared by a paired *t* test (*P < 0.05). F, Effects of CTHRC1 knockdown on the protein expression of EMT markers in HCT‐8 cells treated with TGF‐β or DMSO. The data were compared by a paired *t* test (*P < 0.05). G, Immunofluorescence staining was performed to assess E‐cadherin localization. siCTHRC1 and NC cells were stained with DAPI (blue) for nucleus staining, and E‐cadherin (green) localized in the membrane in cells. H, Downregulation of pSmad2/3 expression in DLD‐1 cells treated with a TGFRI inhibitor (SB‐431542). I, TGF‐β stimulation increases the percentage of pSmad2/3 positive cells in HCT‐8, which was partially blocked by CTHRC1 knockdown. J, Luciferase reporter assays were conducted to quantify Smad2/3 signaling in CTHRC1‐depleted HCT‐8 cells treated with DMSO or TGF‐β. The data were compared by a paired *t* test (*P < 0.05)

To elucidate the prognostic role of CTHRC1 in CRC, OS and DFS were estimated for all CRC patients. The median follow‐up time was 31 months (range 4‐60), the median DFS was 31 months for all patients, and the median OS was 34 months. Fifty‐one patients experienced cancer recurrence, 37 of whom died of cancer progression. The survival analysis revealed that high CTHRC1 expression is associated with poor OS (Figure 1F_1_; *P = *0.001), whereas no significant correlation was observed between CTHRC1 expression and DFS (Figure 1E_1_; *P = *0.127). Intriguingly, we found that high CTHRC1 expression conferred worse DFS (Figure 1E_2_; *P = *0.004) and OS (Figure 1F_2_; *P < *0.001) in the colon cancer cohort, but this association was not observed for DFS (Figure 1E_3_; *P = *0.274) or OS (Figure 1F_3_; *P = *0.345) in the rectal cancer cohort. Consistent with this finding, the data from the THPA database also confirmed the significant association between CTHRC1 mRNA overexpression and poor prognosis in CRC cases, especially in colon cancer cases (Figure 2G_1‐3_).

To evaluate the possibility that CTHRC1 can be used as an independent risk factor for poor prognosis in CRC patients, conventional clinicopathological factors and CTHRC1 protein levels were assessed by Cox's univariate and multivariate hazard regression model. Univariate analyses indicated that CTHRC1 protein expression correlated with DFS only in patients with colon cancer (*P = *0.003, Table S2), whereas CTHRC1 expression was correlated with OS in patients with colorectal (*P = *0.002) or colon cancer (*P = *0.000) but not rectal cancer (Table 2). Multivariate Cox analyses showed that CTHRC1 expression was an independent prognostic factor for OS in all CRC patients (*P = *0.010), especially in colon cancer patients (*P = *0.001, Table 2).

**Table 2:** Univariate and multivariate analyses of overall survival (OS) in CRC patients

	Univariate analysis			Multivariate analysis		
	HR	95% CI	*P* value	HR	95% CI	*P* value
Colorectal						
Age (y)	1.026	1.000‐1.053	0.054			
Gender (male/female)	0.745	0.409‐1.358	0.336			
Location (colon/rectum)	0.465	0.260‐0.832	**0.010**			
Histologic grade (Well/moderate/poor)	2.184	1.115‐4.279	0.023			
Tumor size (<5 cm/≥5 cm)	1.788	1.011‐3.164	0.046			
T stage (T1/T2/T3)	22.698	0.724‐71.988	0.076			
N stage (T1/T2/T3)	2.315	1.616‐3.317	**0.000**			
Duke's stage (A/B/C/D)	4.790	3.082‐7.445	**0.000**	4.823	3.077‐7.561	**0.000**
Venous invasion (absent/present)	1.048	0.414‐2.650	0.921			
Nervous invasion (absent/present)	1.513	0.367‐6.242	0.567			
CTHRC1 expression	2.513	1.406‐4.489	**0.002**	2.158	1.206‐3.861	**0.010**
Colon						
Age (y)	1.016	0.984‐1.049	0.329			
Gender (male/female)	0.952	0.449‐2.017	0.898			
Histologic grade (Well/moderate/poor)	3.095	1.323‐7.237	**0.009**			
Tumor size (<5 cm/≥5 cm)	1.383	0.665‐2.880	0.386			
T stage (T1/T2/T3)	24.178	0.112‐52.196	0.246			
N stage (T1/T2/T3)	2.388	1.485‐3.841	**0.000**			
Duke's stage (A/B/C/D)	3.410	2.011‐5.784	**0.000**	2.377	1.264‐4.467	**0.007**
Venous invasion (absent/present)	4.277	1.804‐10.139	**0.001**	3.927	1.578‐9.774	**0.003**
Nervous invasion (absent/present)	1.641	0.388‐6.947	0.501			
CTHRC1 expression	5.025	2.347‐10.753	**0.000**	4.031	1.771‐9.179	**0.001**
Rectum						
Age (y)	1.028	0.986‐1.073	0.194			
Gender (male/female)	0.534	0.192‐1.482	0.228			
Histologic grade (Well/moderate/poor)	1.331	0.440‐4.031	0.613			
Tumor size (<5 cm/≥5 cm)	2.509	1.017‐6.185	0.046			
T stage (T1/T2/T3)	23.948	0.143‐39.333	0.224			
N stage (T1/T2/T3)	2.317	1.320‐4.067	**0.003**			
Duke's stage (A/B/C/D)	7.142	3.344‐15.257	**0.000**	7.320	3.403‐15.745	**0.000**
Venous invasion (absent/present)	1.827	0.532‐6.277	0.339			
Nervous invasion (absent/present)	2.328	0.537‐10.085	0.259			
CTHRC1 expression	2.001	0.462‐8.666	0.354			

CI, confidence interval; HR, hazard ratio.

Statistical significances (*P* value < 0.05) marked in bold font.

### 3.3 CTHRC1 induces EMT

**Figure 3:**
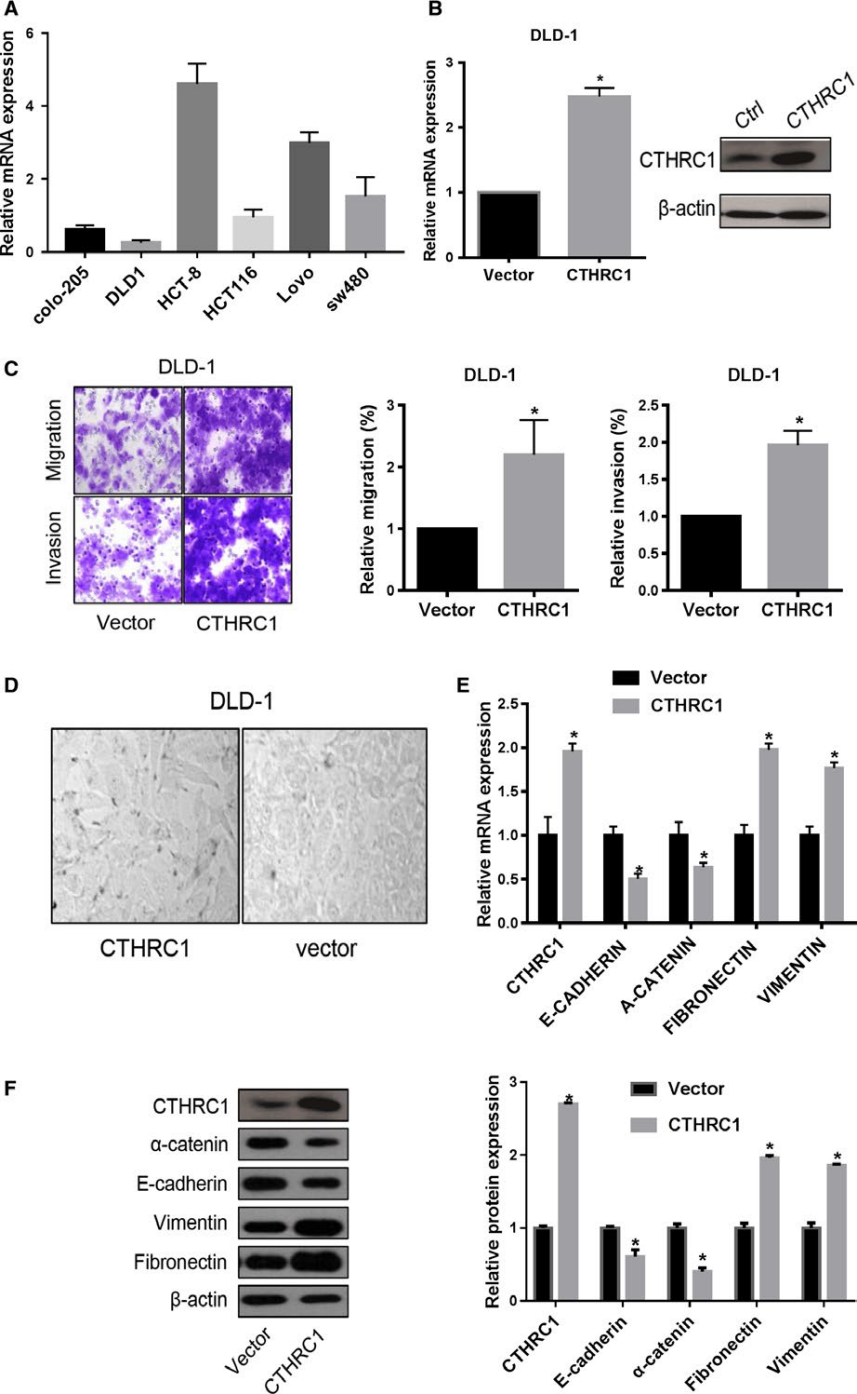
CTHRC1 enhances the metastatic ability of cells and promotes the EMT program in CRC cell lines. A, CTHRC1 expression levels were determined by RT‐qPCR in 6 colon cancer cell lines. The data are presented as the mean ± SD (n = 3). B, DLD‐1 cells were transfected with pIRES2‐EGFP‐CTHRC1 or vector for 48 h, and CTHRC1 mRNA levels in these cells were determined by RT‐qPCR with β‐actin as an internal control. The data were compared by a paired *t* test. C, Ectopic CTHRC1 expression promotes migration and invasion ability in DLD‐1 cells (*P < 0.05). D, Morphological changes after the overexpression of CTHRC1 in DLD‐1 cells. E, The mRNA expression of EMT markers in the CTHRC1 overexpressing DLD‐1 cells determined by RT‐qPCR. The data were compared by a paired *t* test (*P < 0.05). F, The protein expression of EMT markers in the CTHRC1 overexpressing DLD‐1 cells determined by Western blotting. The data were compared by a paired *t* test (*P < 0.05)

The enhanced staining of CTHRC1 in tumor buds suggests a possible role of CTHRC1 in EMT and cancer metastasis. To detect the biological function of CTHRC1 in CRC, we quantified the baseline level of CTHRC1 mRNA in 6 CRC cell lines, including Colo‐205, DLD‐1, HCT‐8, HCT‐116, LoVo, and SW480 cells, by RT‐qPCR assay. The results revealed that DLD‐1 cells exhibited the lowest CTHRC1 level, whereas HCT‐8 cells exhibited the highest CTHRC1 expression level (Figure 3A). In culture, DLD‐1 cells were transfected with the pIRES2‐EGFP‐CTHRC1 (CTHRC1 group) or pIRES2‐EGFP vector (Vector group) for further investigations (Figure 3B). The overexpression of CTHRC1 in DLD‐1 cells led to increased cell migration and invasion (Figure 3C). Histologically, CTHRC1 overexpressing cells underwent an epithelial morphology transition, from a cuboidal‐like appearance to a fibroblastic‐like shape (Figure 3D). This morphological alteration was accompanied by the downregulation of epithelial components (E‐cadherin and α‐catenin) and the upregulation of mesenchymal markers (fibronectin and vimentin) at mRNA level (Figure 3E). Western blotting further confirmed the changes in these EMT markers after CTHRC1 overexpression in DLD‐1 cells, resulting in decreased expression of E‐cadherin and α‐catenin and increased production of fibronectin and vimentin (Figure 3F). Taken together, these data suggest that CTHRC1 enhances CRC migration and invasion by inducing EMT.

### 3.4 Effect of TGF‐β on the regulation of CTHRC1 expression

TGF‐β signaling is a well‐known signaling pathway that activates EMT and promotes metastasis during tumorigenesis. To investigate whether CTHRC1 executes its function via TGF‐β signaling, we treated DLD‐1 cells with 50 ng/mL TGF‐β in culture medium and observed that TGF‐β‐treated cells presented a gradual increase in CTHRC1 expression (Figure 2A). In contrast, we knocked down CTHRC1 expression in HCT‐8 cells using siRNA (Figure 2B). These CTHRC1‐silenced cells were then treated with recombinant TGF‐β for 5 days. We found that silencing CTHRC1 expression partially blocked the morphological transition induced by TGF‐β treatment (Figure 2C). In addition, TGF‐β‐induced migration and invasion were partially attenuated by CTHRC1 knockdown (*P* < 0.05, Figure 2D). The effect of TGF‐β treatment on epithelial markers (E‐cadherin and α‐catenin) and mesenchymal markers (fibronectin and vimentin) was also partially recovered by siCTHRC1 (*P* < 0.05, Figure 2E). Western blotting analyses further confirmed these findings (Figure 2F). Furthermore, the immunofluorescence staining results showed that CTHRC1 knockdown accompanied by TGF‐β treatment prevented the disappearance of E‐cadherin in cell‐cell junctions (Figure 2G).

The regulatory effect of TGF‐β on EMT was mediated mainly by Smad2/3 signaling. As expected, we found that phosphorylated Smad2/3 (pSmad2/3) levels were obviously decreased in DLD‐1 cells treated with the TGFRI inhibitor SB‐431542 compared with those of the untreated control (Figure 2H). Moreover, the percentage of pSmad2/3‐positive cells was dramatically increased in the TGF‐β‐treated HCT‐8 cells, and this effect was partially blocked by CTHRC1 knockdown (Figure 2I). In addition, luciferase reporter assays were conducted to quantify the effects of CTHRC1 on Smad2/3 activation. Notably, Smad2/3 signals were obviously deceased in CTHRC1‐depleted HCT‐8 cells compared with those in the corresponding control cells (Figure 2J). The induction of Smad2/3 signaling by TGF‐β was significantly hampered by CTHRC1 knockdown, suggesting an important role of CTHRC1 in TGF‐β‐induced EMT.

## 4 DISCUSSION

CTHRC1 was initially described as a glycosylated 28‐kDa protein secreted during the injury‐repair process.^9^ The overexpression of CTHRC1 has been demonstrated to act as a key regulator of cell migration by reducing collagen matrix deposition in injured arteries.^9^ While physiological CTHRC1 expression is essential for wound healing, the pathological reactivation of CTHRC1 drives human tumor development. Indeed, CTHRC1 is highly expressed in most human solid tumors.^14^, ^20^, ^21^ Consistent with these discoveries, we observed that CTHRC1 expression was dramatically increased in CRC samples compared to the expression levels in normal mucosa. These findings were further confirmed with TCGA and THPA CRC data. These results suggested that the functions of CTHRC1 are associated with CRC development.

Some investigators reported that upregulated CTHRC1 acts as a prognostic factor in melanoma^14^ and breast cancer.^22^ In addition, Tan et al demonstrated that increased expression of CTHRC1 occurs in peritoneal carcinomatosis of CRC and predicts prognosis in CRC patients.^15^ Our results verified that the overexpression of CTHRC1 in CRC patients results in poor outcomes. Furthermore, the multivariable regression models revealed that CTHRC1 expression is an independent prognostic predictor in CRC. Interestingly, CTHRC1 upregulation has better prognostic value for colon cancer than for rectal cancer, which may shade new light on precise postoperative management of CRC patients.

EMT progression involves the localized occurrence of a loss of epithelial differentiation and the acquisition of a mesenchymal phenotype. This process enhances the motility and invasion of cancer cells and plays an important part in the consensus molecular subtype (CMS) classifications.^23^ CRCs with EMT signature are classified into CMS4 subgroup (an EMT‐related subtype), characterized as more aggressive phenotype showing poorer overall survival and signatures of high TGF‐β signaling and EMT activation.^7^ Tumor budding, with single cells or clusters of up to five cells detached from the main tumor mass, is the morphologic manifestation of EMT. Tumor budding at the advancing front of CRC is an early event in the metastatic process.^24^ This phenomenon can predict patients at high risk of recurrence and serve as an independent prognostic marker in CRC patients.^25^, ^26^ In our studies, CTHRC1 expression in tumor buds revealed a more aggressive phenotype of CRC; our results suggested that CTHRC1 may be a tumor invasion promoter. In in vitro experiments, we confirmed that CTHRC1 enhances the metastatic ability of CRC cells and promotes the EMT process at the transcriptional and translational levels. Consistently, Hou et al found that CTHRC1 promotes EMT in ovarian cancer.^21^ Jin et al reported that the knockdown of CTHRC1 inhibits EMT in renal cell carcinoma.^27^ Taken together, these data suggested that CTHRC1 might play an extensive oncogenic role by inducing the process of EMT and thus promote CRC cells invasion and migration.

Several signaling pathways are involved in the steps of CTHRC1 overexpression. Yamamoto et al reported that CTHRC1 activated the Wnt/PCP pathway by stabilizing the Wnt‐receptor complex and suppressing the canonical Wnt pathway.^28^ CTHRC1 could promote the proliferation and invasiveness of colorectal cancer cell by activating Wnt/PCP signaling.^29^ In hepatocellular carcinoma, CTHRC1 knockdown suppressed cell migration/invasion and induced apoptosis via integrin β downregulation.^30^ Kim et al^16^ proved that CTHRC1 could activate the Src and Erk signaling cascades and upregulate matrix metalloproteinase 9 (MMP9), thus promote colorectal cancer cell invasion. The TGF‐β pathway is also an important participant in this complicated mechanism. It has been reported that CTHRC1 levels are enhanced in fibroblasts and chondrocytic cells in response to TGF‐β family members,^10^ and many lines of evidence indicated that CTHRC1 regulates the TGF‐β signaling cascade via the activation of Smad2/3 phosphorylation during arterial injury^9^, ^31^ and hepatic fibrosis.^32^ We demonstrated that treatment with recombinant TGF‐β increases the CTHRC1 level in CRC cells, resulting in invasiveness and EMT promotion, by activating the TGF‐β signaling pathway. In addition, our results revealed that Smad2/3 signals were obviously deceased in CTHRC1‐depleted cells, and Smad2/3 signals were significant hampered by CTHRC1 knockdown. These findings show the ability of CTHRC1 to activate TGF‐β signaling through Smad2 and Smad3 and contribute to the EMT process and CRC metastasis.

The data presented by us show that the overexpression of CTHRC1 is associated with EMT process and involves in TGF‐β activation in CRC cells. These findings suggest that CTHRC1 is implicated in the molecularly specific subtype, such as CMS4, which provides a perspective evaluating the potential value of CTHRC1 in the molecular classification of CRCs for further investigations. In addition, the underlying mechanism of CTHRC1 in EMT process is limited, more experimental approaches are needed to provide mechanistic insights into the cross talk between CTHRC1 and TGF‐β signaling pathways. Finally, further studies and larger case series with long‐term follow‐up are necessary to assess the validity and durability of the prognostic value of the CTHRC1 expression.

## 5 CONCLUSION

In summary, our observations indicated that CTHRC1 is overexpressed in CRC and can be utilized as an independent prognostic predictor in CRC patients. Furthermore, CTHRC1 drives the pathogenesis of the EMT process in CRC by activating the TGF‐β pathway. Since EMT is a critical step toward invasion and metastasis, our results suggest that CTHRC1 has potential as a therapeutic target for disrupting CRC progression.
