# Supplementary material for: CTHRC1 overexpression predicts poor survival and enhances epithelial‐mesenchymal transition in colorectal cancer
Source: Cancer Med. 2018 Oct 9;7(11):5643–54. doi: 10.1002/cam4.1807 (PMC6247052; doi:10.1002/cam4.1807)
Supplement: Supplementary file 1 [file CAM4-7-5643-s001.docx]

**Supplementary Materials and Methods**

**Patients and Follow-up**

A data set from *The Cancer Genome Atlas* (TCGA) was utilized for the evaluation of CTHRC1 mRNA expression in CRC. The data from 224 CRC and 22 adjacent normal tissue samples are available online (https://genome-cancer.ucsc.edu/proj/site/hgHeatmap/). Another data set from *The Human Protein Atlas* (THPA) was utilized for the evaluation of CTHRC1 protein expression as a prognostic factor for CRC. The CTHRC1 expression data for 597 CRC and adjacent normal tissues (196 transverse mucosa and 149 normal sigmoid mucosa samples), as well as the survival analysis results for 597 CRC tissue samples, are available at the Human Protein Atlas (<http://www.proteinatlas.org/)>.

**Cell Culture and Treatment**

Cells were grown in Roswell Park Memorial Institute (RPMI) 1640 medium (Gibco BRL, NY, USA) for DLD-1 cells or Dulbecco’s modified Eagle’s medium (DMEM) (Gibco BRL, NY, USA) for HCT-8 cells. Media were supplemented with 10% fetal bovine serum (FBS) (Gibco BRL, NY, USA) and 1% penicillin/streptomycin (Gibco BRL, NY, USA), and cells were cultured at 37°C in a humidified atmosphere of 5% CO2.

**Migration and Invasion Assays**

Cell migration was assessed using a QCMTM 24-well Cell Migration Assay kit (Millipore, MA, USA). Cells (2×10^4^) suspended in serum-free medium were placed in the top chamber, and medium containing 10% FBS was placed in the lower chamber. The cells were incubated for 12 h at 37°C. Nonmigrating cells on the upper surface of the membrane were gently removed. The migrated cells were then fixed with 95% ethanol and stained with crystal violet. The stained cells were photographed under an inverted light microscope and counted manually using five randomly selected areas. The migration assays were performed in triplicate. For the cell invasion assay, the membranes of the upper chambers were precoated with 1 mg/mL Matrigel.

**Western Blot Analysis**

Protein samples (60 µg each) were separated by 10% sodium dodecyl sulfate-polyacrylamide gel electrophoresis (SDS-PAGE) and then electrotransferred onto nitrocellulose membranes (Millipore, MA, USA). The membranes were then exposed to primary and secondary antibodies at the optimum dilutions, and immunoreactive signals were detected with the Immobilon Western Chemiluminescent HRP Substrate (Millipore).

Supplementary Table1: The primers in RT-qPCR

| Genes | Sequences |
| --- | --- |
| CTHRC1 | F: TGGTATTTCACATTCAATGGAGCTG |
|  | R: TGGGTAATCTGAACAAGTGCCAAC |
| E-cadherin | F: tggaggaattcttgctttgrc |
|  | R: CGTACATGTCAGCCAGCTTC |
| α-catenin | F: GAGCCAGTTTCTCAAGGAGGA |
|  | R: CGTCGATCTC CTTAGGACGTC |
| β-catenin | F: GCCAAGTGGGTGGTATAGAG |
|  | R: GCTGGGTATCCTGATGTGC |
| γ-catenin | F: GAGAGTGTGCTGAAGATTCTG |
|  | R: TGATGTCGTCCTTGTCACC |
| Fibronection | F: CCTTAAGCCTTCTGCTCTGG |
|  | R: CGGCAAAAGAAAGCAGAACT |
| Vimentin | F: CGCTTCGCCAACTACAT |
|  | R: AGGGCATCCACTTCACAG |
| N-cadherin | F: CACTGCTCAGGACCCAGAT |
|  | R: TAAGCCGAGTGATGGTCC |
| β-actin | F: atctggcaccacaccttctac |
|  | R: CAGCCAGGTCCAGACGCAGG |

Supplementary Table 2 Univariate and multivariate analyses of disease-free survival (DFS) in CRC patients

|  | Univariate analysis | | | | multivariate analysis | | | |
| --- | --- | --- | --- | --- | --- | --- | --- | --- |
|  | HR | | 95% CI | P value | HR | 95% CI | P value | |
| **Colorectal** | | | | | | | | |
| Age (years) | 1.023 | | 0.998-1.049 | 0.075 |  |  |  | |
| Gender  (male/female) | 0.760 | | 0.423-1.365 | 0.359 |  |  |  | |
| Location (colon/rectum) | 0.467 | | 0.265-0.825 | **0.009** |  |  |  | |
| Histologic grade (Well/moderate/poorly) | 2.360 | | 1.232-4.520 | **0.010** |  |  |  | |
| Tumor size (<5cm/>=5cm) | 1.820 | | 1.041-3.182 | **0.036** |  |  |  | |
| T stage (T1/T2/T3) | 7.983 | 1.113-57.237 | | **0.039** |  |  |  |  |
| N stage (T1/T2/T3) | 2.046 | 1.446-2.894 | | **0.000** |  |  |  |  |
| Duke’s stage (A/B/C/D) | 12.598 | 7.089-22.388 | | **0.000** | 12.598 | 7.089-22.388 | **0.000** |  |
| Venous invasion (absent/present) | 1.306 | | 0.556-3.070 | 0.540 |  |  |  | |
| Nervous invasion (absent/present) | 0.673 | | 0.093-4.878 | 0.695 |  |  |  | |
| CTHRC1 expression | 0.578 | | 0.319-1.047 | 0.071 |  |  |  | |
| **Colon** | | | | | | | | |
| Age (years) | 1.011 | | 0.981-1.043 | 0.466 |  |  |  | |
| Gender  (male/female) | 0.769 | | 0.360-1.643 | 0.497 |  |  |  | |
| Histologic grade (Well/moderate/poorly) | 1.873 | | 0.741-4.731 | 0.185 |  |  |  | |
| Tumor size (<5cm/>=5cm) | 1.438 | | 0.701-2.950 | 0.322 |  |  |  | |
| T stage (T1/T2/T3) | 24.185 | 0.137-42.470 | | 0.228 |  |  |  |  |
| N stage (T1/T2/T3) | 2.132 | 1.339-3.396 | | **0.001** |  |  |  |  |
| Duke’s stage (A/B/C/D) | 6.883 | 3.582-13.224 | | **0.000** | 7.454 | 3.758-14.784 | **0.000** |  |
| Venous invasion (absent/present) | 3.345 | | 1.350-8.290 | **0.009** | 3.146 | 1.219-8.120 | **0.018** | |
| Nervous invasion (absent/present) | 1.396 | | 0.330-5.902 | 0.650 |  |  |  | |
| CTHRC1 expression | 2.974 | | 1.134-7.804 | **0.003** |  |  |  | |
| **Rectum** | | | | | | | | |
| Age (years) | 1.030 | | 0.988-1.073 | 0.163 |  |  |  | |
| Gender  (male/female) | 0.790 | | 0.315-1.979 | 0.615 |  |  |  | |
| Histologic grade (Well/moderate/poorly) | 3.092 | | 1.201-7.963 | **0.019** |  |  |  | |
| Tumor size (<5cm/>=5cm) | 1.456 | | 0.595-3.566 | 0.411 |  |  |  | |
| T stage (T1/T2/T3) | 3.911 | 0.538-28.461 | | 0.178 |  |  |  |  |
| N stage (T1/T2/T3) | 2.120 | 1.234-3.644 | | **0.007** |  |  |  |  |
| Duke’s stage (A/B/C/D) | 39.323 | 11.403-35.605 | | **0.000** | 39.323 | 11.403-35.605 | **0.000** |  |
| Venous invasion (absent/present) | 0.531 | | 0.071-3.972 | 0.538 |  |  |  | |
| Nervous invasion (absent/present) | 0.046 | | 0.000-39.667 | 0.511 |  |  |  | |
| CTHRC1 expression | 2.344 | | 0.544-10.109 | 0.253 |  |  |  | |

HR = hazard ratio; CI = confidence interval
